# Improving Survey Response Rates from Parents in School-Based Research Using a Multi-Level Approach

**DOI:** 10.1371/journal.pone.0126950

**Published:** 2015-05-11

**Authors:** Elizabeth J. Schilpzand, Emma Sciberras, Daryl Efron, Vicki Anderson, Jan M. Nicholson

**Affiliations:** 1 Murdoch Childrens Research Institute, Parkville, VIC Australia; 2 The Royal Children’s Hospital, Parkville, VIC Australia; 3 The University of Melbourne, Parkville, VIC Australia; 4 Judith Lumley Centre, La Trobe University, Melbourne, VIC Australia; 5 The Parenting Research Centre, Melbourne, VIC Australia; Leibniz Institute for Prevention Research and Epidemiology (BIPS), GERMANY

## Abstract

**Background:**

While schools can provide a comprehensive sampling frame for community-based studies of children and their families, recruitment is challenging. Multi-level approaches which engage multiple school stakeholders have been recommended but few studies have documented their effects. This paper compares the impact of a standard versus enhanced engagement approach on multiple indicators of recruitment: parent response rates, response times, reminders required and sample characteristics.

**Methods:**

Parents and teachers were distributed a brief screening questionnaire as a first step for recruitment to a longitudinal study, with two cohorts recruited in consecutive years (cohort 1 2011, cohort 2 2012). For cohort 2, additional engagement strategies included the use of pre-notification postcards, improved study materials, and recruitment progress graphs provided to school staff. Chi-square and t-tests were used to examine cohort differences.

**Results:**

Compared to cohort 1, a higher proportion of cohort 2 parents responded to the survey (76% versus 69%; p < 0.001), consented to participate (71% versus 56%; p < 0.001), agreed to teacher participation (90% versus 82%; p < 0.001) and agreed to follow-up contact (91% versus 80%; p < 0.001). Fewer cohort 2 parents required reminders (52% versus 63%; p < 0.001), and cohort 2 parents responded more promptly than cohort 1 parents (mean difference: 19.4 days, 95% CI: 18.0 to 20.9, p < 0.001).

**Conclusion:**

These results illustrate the value of investing in a relatively simple multi-level strategy to maximise parent response rates, and potentially reduce recruitment time and costs.

## Introduction

Approximately 98.8% of Australian children aged 6–15 years are enrolled in school, [1] making the school setting appropriate for ascertaining population-representative samples of children and their parents. Numerous studies have demonstrated that school-based recruitment is challenging [2–5] and it can be difficult to achieve the high participation rates crucial for ensuring the generalizability of research findings. Suboptimal recruitment can extend recruitment periods, presenting budgetary and logistic challenges. Numerous recommendations have been made for increasing sample response rates generally [6, 7]. However, in the school setting, few studies have documented strategies that improve response rates or have compared the effectiveness of alternate recruitment approaches. The current study addresses this gap by comparing school-based recruitment approaches in a large sample of elementary school aged children.

In order to reach parents (or guardians) through schools, researchers need to engage and maintain the goodwill and participation of a variety of stakeholders including, but not restricted to, government education agencies, district representatives, school principals, teaching and administrative staff, children and interested community members [4, 5]. While the extent to which the school community engages with and supports the research is likely to influence parents’ decisions around participation, no studies have formally examined how the intensity of school engagement influences parent response rates. The complexities inherent in multi-level recruitment present a challenge for school-based health researchers to identify and implement optimal recruitment methods.

A number of studies have described methods to improve participant response rates in school-based health studies, [4, 8] however, the majority have used non-experimental designs, provided little detail about their response rate strategies and failed to compare the effectiveness of different approaches to improving response rates. Wolfenden et al. (2009) reviewed the literature examining the effectiveness of strategies for obtaining active parental consent through schools. Their review suggested five key strategies for enhancing parent participation rates: 1) promoting the research to school principals, teachers, parents and students [2, 9]; 2) dissemination of study information using methods allowing direct rather than mediated communication (i.e., face-to- face contact with parents was more effective than receiving information via students) [10]; 3) offering incentives to teachers, peers and individual participants [11, 12]; 4) providing three follow-up reminder contacts to parents who have not made a decision regarding participation [11]; and 5) ensuring that a dedicated member of the research team co-ordinates and closely monitors the recruitment process (i.e., a dedicated study coordinator is more effective than leaving responsibility to teachers) [11]. This review suggests that in addition to targeting parents through direct communication and targeted follow-ups, the successful engagement of other members of the school community may play a role in promoting parent participation in research.

We examined the impact of an enhanced engagement approach on parent recruitment as part of the Children’s Attention Project. At baseline the study involved collecting brief parent and teacher data on all Grade 1 children (the second year of formal schooling in Australia), with recruitment occurring over two consecutive years (cohorts 1 and 2 respectively). Our goal was to identify how the engagement strategies employed for cohort 1 could be improved for cohort 2, and to evaluate impact on a range of indicators of parent response and recruitment rates. Specifically, we sought to compare the impact of the enhanced research condition (cohort 2) relative to the standard condition (cohort 1) on parent response rates, response times, reminders required, consent rates (for teacher participation and for later follow-up) and sample characteristics. Our enhanced methodology aimed to provide more frequent, direct, personalised contact with parents, combined with greater involvement of multiple stakeholders, including the Education Department, principals, teaching staff, administrative staff, and parents/primary caregivers. We hypothesised that compared to the standard condition, the enhanced method would result in higher parent response and consent rates, faster response times, fewer reminders and the recruitment of a more representative sample. Teacher responses rates were not directly targeted in the enhanced approach as these were very high for cohort 1 (98.7%). Nonetheless, we compared teacher response rates, response times, and reminders required for the two cohorts to assess whether these were maintained or adversely affected by the enhanced parent engagement approach.

## Methods

### Study design

This study, approved by the Human Research Ethics Committees of the Royal Children’s Hospital (#31056) and the Victorian Department of Education and Early Childhood Development (#2011_001095), was conducted as part of the Children’s Attention Project (CAP). Parents (or guardians) were asked to nominate their consent on the returned survey and provide consent for their child’s teacher to also complete a screening survey. This method of consent was approved by the ethics committee. CAP is a community-based longitudinal study of children with ADHD and non-ADHD controls that seeks to examine the developmental course of ADHD and the factors influencing outcomes. As a first step in identifying the sample for the longitudinal study, we sought to screen all children in Grade 1 at participating state government elementary schools using parent- and teacher-reported symptoms of ADHD. All children meeting pre-determined criteria on the combination of parent and teacher screeners (ADHD group), plus a matched sample of children who did not meet criteria on both screeners (non-ADHD group), were invited to participate in the next stage of the research (formal confirmatory diagnostic assessment of ADHD status, followed by baseline data collection from parents, children and teachers). The study’s aims, key research questions and design have been detailed previously [13].

Elementary schools were recruited to the study via the state authority responsible for government schools (Victorian Government Department of Education and Early Child Development). Schools in two regions (Eastern and Western) of metropolitan Melbourne, Australia, were selected to ensure coverage of diverse socioeconomic communities [14]. Forty-one schools participated in both 2011 (cohort 1 recruitment) and 2012 (cohort 2 recruitment) with an additional 2 schools who had heard about the study asking to participate in 2012.

### Data collection

The study sought to collect screening data from the parents and classroom teachers of a representative sample of Grade 1 students in 2011 (cohort 1) and 2012 (cohort 2), with a goal of achieving parent and teacher response rates for over 70% of all students. Data collection took place in the third and fourth terms of the academic year for cohort 1 (June—October) and in the second and third terms of the academic year for cohort 2 (April—August). Schools provided the researchers with a list of first names, gender and postcodes for all Grade 1 students. Parents were distributed information packs inviting them to participate by completing a screening survey and providing consent for their child’s teacher to also complete a screening survey. Parent and teacher screeners each contained the 10-item Conners 3 ADHD Index [15]. Parent screeners also asked whether the child had a previous diagnosis of with ADHD and collected brief demographic details (e.g., child date of birth and gender; language spoken at home) and contact details for subsequent follow-up. Neighborhood socioeconomic disadvantage was measured by the Socio-Economic Indexes for Areas Disadvantage Index (SEIFA) [16] for the child’s postcode of residence (mean (SD) = 1000 (100); higher scores reflect less disadvantage). On the basis of combined parent and teacher screening data, a sub-sample of parents was then approached to participate in a detailed baseline assessment and the longitudinal study (involving parent, teacher and child participation). The following sections and Table 1 describe the processes used to maximise parent and teacher participation rates in screening and consent to the longitudinal study.

**Table 1:** Multi-level approach to improving response rates.

Strategy			C1	C2	Goal/s	Target
*School Recruitment*						
1.	Presentations at regional network meetings		✓	✓	Establish credibility of the research team and utility of the research findings	Education Department, Principal
						Make case for why this research is important in general, and of value to the school	
						Make connections with schools interested in participating in the research	
2.	Meetings with school principals					Principal
		a)	Provide information package and copies of study materials	✓	✓	Establish personal links with schools	
		b)	Ask principal to sign a school research agreement committing to participating in the research	✓	✓	Make the research requirements clear	
		c)	Request a school liaison officer is nominated for study related correspondence	✓	✓	Provide opportunity to answer questions and address concerns	
		d)	Provide timeline for the project and time commitments from staff	✓	✓	Minimise administrative burden	
3.	Meeting with Grade 1 teachers				Ensure support of teachers for school’s participation	Teaching staff
		e)	Provide information pack and copies of study materials	✓	✓	Provide opportunity for questions or concerns to be raised	
4.	School engagement					Education Department, Principal, Teaching staff
		a)	Provide relief funding in recognition of staff efforts	✓	✓	Acknowledge efforts of school staff	
		b)	Undertake any requested speaking engagements	✓	✓		
		c)	Provide professional development opportunity to teachers	✓	✓		
		d)	Provide quarterly newsletters	✓	✓		
*Pre-distribution Strategies*						
1.	Presentations at regional network meetings and school staff meetings		✓	✓	Engage school staff and inform them of the research	Education Department, Principal, Teaching staff
						Maintain communication and visibility	
						Provide opportunity for questions and response to concerns	
						Demonstrate utility of the research findings and benefits of participation	
2.	Pre-notification of survey distribution:					Teaching staff, Administrative staff, Parents/Primary Caregivers
		a)	Emails to schools liaisons	✓	✓	Ensure parents and school staff were aware of study commencement	
		b)	School newsletter advertisements	✓	✓	Maintain communication and visibility	
		c)	Pre-notification postcard to parents		✓	Minimise opportunities for adverse parent reactions	
		d)	Study posters in the school		✓	Maximise response rates	
3.	Incentive for participation					Principal, Teaching staff, Administrative staff, Parents/Primary Caregivers
		a)	School prize for school within the region with highest response rate	✓	✓	Maximise response rates	
*Parent Strategies*						
1.	Information packages for school liaisons and teachers			✓	Establish personal link with teaching staff	Teaching staff, Administrative staff
						Minimise administrative burden for school staff	
2.	Survey Materials					Parents/Primary Caregivers
		a)	Provide reply-paid envelopes	✓	✓	Maximise response rates	
		b)	Short survey with coloured cover page, attractive layout and presentation	✓	✓	Demonstrate school support of research and compatibility with school environment	
		c)	Personalised envelopes and letters with child’s name and school logo		✓	Minimise opportunities for adverse parent reactions	
		d)	Cover letter with a study logo and school logo, co-signed by school principal		✓	Minimise costs of materials	
		e)	Cover letters personally signed by a research staff member in addition to being co-signed by the school principal		✓		
		f)	Provide parents with the option to opt-out		✓		
		g)	Simplify survey materials by combining consent form and survey		✓		
		h)	Assure confidentiality		✓		
3.	Survey Distribution					Teaching staff, Parents/Primary Caregivers
		a)	Send information to parents through the school	✓	✓	Maintain communication and visibility	
		b)	Research assistant explain survey to children in the classroom	✓	✓	Maximise opportunities to participate	
		c)	Colourful collection box in each classroom for returned surveys	✓	✓	Minimise administrative burden for school staff	
4.	Survey Reminders					Parents/Primary Caregivers
		a)	Provide personalised reminder letter to parents	✓	✓	Demonstrate school support of research and compatibility with school environment	
		b)	Advertisement reminder in school newsletter	✓	✓	Maximise opportunities to participate	
5.	Progress Update to Schools				Maintain communication and visibility	Principal, Teaching staff, Administrative Staff
		a)	Graphs demonstrating response rate by school		✓	Maximise opportunities to participate	
		b)	Fortnightly email to school liaisons and teachers with an update on response rates		✓	Acknowledge efforts	
		c)	Graphs demonstrating response rates by classrooms within the school		✓	Opportunity to problem solve barriers to participation	
6.	Progress Update to Teachers				Acknowledge efforts of teachers	Principal, Teaching staff
		a)	Provide teachers with a list of children with outstanding forms		✓	Opportunity to problem solve barriers to participation	
*Teacher Strategies*						
1.	Distribute surveys to teachers in batches		✓	✓	Minimise administrative burden	Teaching staff
2.	Reminders and replacement surveys sent after two weeks		✓	✓	Maximise opportunities to participate	Teaching staff

### School recruitment strategies

Strategies employed to recruit schools were consistent across both waves of recruitment. As shown in Table 1, at the school level, strategies were targeted at engagement of Education Department representatives, school principals and classroom teachers. These included researcher presentations at regional meetings of school principals, followed by individual meetings with interested principals and separately with their Grade 1 teachers to discuss project details and address any concerns raised. Schools were accepted into the study if teachers indicated support and principals signed a research agreement supporting the school’s participation. Teacher relief funding was provided to schools to compensate for teacher participation.

### Standard condition parent and teacher recruitment strategies

Strategies to engage parents and teachers were structured to draw attention to the study prior to data collection (Table 1 Pre-distribution strategies), and to encourage participation during the data collection period (Table 1 Parent strategies; Teacher strategies). Parent screening surveys were sent home with children through the school and returned in sealed envelopes via classroom collection boxes or reply paid mail. Reminder letters and replacement surveys were sent to parents who did not opt-out and did not return the survey at two and four weeks after the initial distribution.

Upon receipt of parental consent, teachers were asked to complete the teacher screening survey. To streamline workloads for teachers, their screeners were sent in batches, so that teachers completed these for the majority of children in their class at one time. Reminders and replacement surveys were sent after two weeks.

### Enhanced condition parent and teacher recruitment strategies

After completion of cohort 1 recruitment and data collection, feedback was sought from school staff on strategies that could be used to enhance recruitment for cohort 2. As shown in Table 1 the enhanced approach was targeted at multiple stakeholders: Education Department, principals, teaching staff, administrative staff, and parents/primary caregivers. To ensure parents were aware of study commencement and to minimise adverse reactions arising from receiving study information without prior warning, a pre-notification postcard was mailed directly to parents from the school prior to survey distribution. Parent materials were also modified to be more personalised and attractive (e.g., hand signed letters, study logo). To maintain study visibility and encourage schools to actively seek ways to promote the return of parent and teacher surveys (whether completed or returned blank with a decline to participate), school staff were provided with graphs documenting the study’s progress in their school throughout the data collection period. These were sent to principals, teaching and administrative staff, and provided a comparison of their response rate to other schools within their region, and a profile of response rates by classroom at their school.

### Statistical analyses

Chi-square and t-tests were used to examine differences in response rates, response times and reminder rates between cohort 1 and cohort 2 and sample characteristics between responders and non-responders. Analyses were conducted using Stata 12.1 (Stata Corp, College Station, TX, USA).

## Results

### Sample characteristics

For cohort 1, responses were received from the parents of 1891 children (69.2%), with screening data provided by 1545 parents (56.5%) and the teachers of 1502 children (98.7% of those for whom consent was provided). Complete parent and teacher screening data were available for 55% of the potential cohort 1 population. For cohort 2, responses were received from the parents of 2561 children (76.1%), with screening data provided by 2401 parents (71.3%) and the teachers of 2249 children (99.3% of those for whom consent was provided). Complete parent and teacher screening data were available for 66.7% of the potential cohort 2 population.

Compared to cohort 1, children in cohort 2 were somewhat younger as they were recruited earlier in the year to the previous cohort. Cohort 2 participants were from more socially disadvantaged areas compared to cohort 1 participants (mean 1013.0 vs 1016.3)—see Table 2.

**Table 2:** Response rate and sample characteristics for cohort 1 and 2.

	Cohort 1	Cohort 2	p value
	n = 2733^a^	n = 3367^a^	
Participating schools, n	41	43	
Participating teachers, n	201	225	
*Sample Characteristics*			
Child age, mean (SD)	6.8 (0.4)	6.6 (0.4)	<0.001
Male, n (%)	1402 (52.5)	1710 (51.1)	0.28
Social advantage ^b^	1016.3 (43.7)	1013.0 (43.4)	0.003
*Parent Screeners*			
Response received ^c^, n (%)	1891 (69.2)	2561 (76.1)	<0.001
Days to return survey, mean (SD)	40.1 (33.2)	20.7 (15.2)	<0.001
Completed survey, n (%)	1545 (56.5)	2401 (71.3)	<0.001
Consented to teacher participation, n (%)	1524 (81.8)	2291 (90.2)	<0.001
Required reminder, n (%)	1715 (62.8)	1740 (51.7)	<0.001
Required second reminder, n (%)	1264 (46.3)	1025 (30.4)	<0.001
*Teacher Screeners*			
Completed survey, n (%)	1502 (98.7)	2249 (99.3)	0.04
Days to return survey, mean (SD)	22.4 (26.6)	26.3 (31.0)	<0.001
Requiring reminder, n (%)	272 (10.0)	942 (28.0)	<0.001
Parent consented to be contacted for further follow-up, n (%)	1507 (79.7)	2327 (90.9)	<0.001
Complete parent and teacher data, n (%)	1503 (55.0)	2247 (66.7)	<0.001
*Longitudinal Study Participation*			
Eligible for recruitment into longitudinal study, n (%)	379 (97.4)	418 (92.5)	0.001
Recruited into longitudinal study, n (%)	246 (64.7)	252 (60.3)	0.20

^a^ n = number of parents approached to participate

^b^ Socio Economic Indexes for Areas Disadvantage

^c^ ‘Response received’ includes parents who responded indicating they did not consent to participation

Note: Response rates may differ to other papers reporting on the Children’s Attention Project sample. Other papers reporting on this sample report slightly lower response rates due to exclusionary criteria applied post the return of the baseline screening surveys

### Response rates differences across cohort 1 and cohort 2

As shown in Table 2, compared to parents in cohort 1, parents in cohort 2 were more likely to respond to the screening survey (76% versus 69%; p < 0.001), consent and complete the screening survey (71% versus 57%; p < 0.001), agree to teacher participation (90% versus 82%; p < 0.001) and consent to further follow-up (91% versus 80%; p < 0.001). Fewer cohort 2 parent participants received a reminder to return their surveys (52% versus 63%; p < 0.001), and on average they took less time to respond to the survey (mean difference: 19.4 days, 95% CI: 18.0 to 20.9, p < 0.001). While the proportion of children with complete parent and teacher screening data was higher for cohort 2 than cohort 1 (67% versus 55%; p<.001), there was no difference between cohorts in the number of participants recruited into the longitudinal study (65% vs 60%; p = 0.20). We received more teacher surveys for children in cohort 2, compared to those in cohort 1 (99.3% vs 98.7%, p = 0.04, however these were returned at a slower rate (mean difference: -3.9 days, 95% CI: -5.8 to -2.0, p <0.001) and required a reminder (28% vs 10%, p < 0.001).

### Difference between participants and non-participants across the cohorts

For both cohorts, participants (parents who completed the screening survey) did not differ from non-participants with respect to child age (see Table 3). Parents who participated were more likely to come from socially advantaged areas. Compared to non-participants, participants in cohort 2 were more likely to be parents of female children.

**Table 3:** Sample characteristics of participants and non-participants in cohort 1 and cohort 2.

	Cohort 1			Cohort 2		
	Participants^a^	Non-Participants	p value	Participants^a^	Non-Participants	p value
	N = 1545	N = 1188		N = 2401	N = 966	
Child age, mean (SD)	6.8 (0.4)	6.8 (0.4)	0.54	6.6 (0.4)	6.6 (0.4)	0.66
Male, n (%)	815 (54.0)	587 (50.5)	0.07	1194 (49.8)	516 (54.4)	0.02
Social advantage ^b^	1021.7 (43.4)	1009.4 (43.1)	<0.001	1016.8 (42.6)	1002.3 (43.7)	<0.001

^a^ Participants are parents who completed the screening survey

^**b**^ Socio Economic Indexes for Areas Disadvantage

## Discussion

We examined the effectiveness of an enhanced research condition targeted at multiple stakeholders (Education Department staff, principals, teaching staff, administrative staff and parents) compared to a standard research condition in improving parent response rates. As expected, when compared to the standard condition (cohort 1), parents in the enhanced condition (cohort 2) were more likely to respond to surveys, consent to participate, agree to teacher participation, require less reminders, and respond more promptly to the survey. Our results support the value of investing in a multi-level strategy to maximise response rates in school-based research. The higher and more rapidly achieved parent response rate for cohort 2 resulted in savings in time and resources and significant cost savings to the project.

The effectiveness of the enhanced approach to recruitment highlights the impact that relatively simple strategies can have on parents’ participation in research. The main parent-focussed strategies in the enhanced research condition were pre-notification postcards alerting parents to the forthcoming survey and improved study materials (e.g., inclusion of study logo, hand signed letters). The main strategies targeting school staff were progress graphs documenting the school’s response rates. Together these additional strategies resulted in a 9% increase in the numbers of parents responding to the survey, a 15% increase in parent consent and an 8% increase in parents’ providing consent for teacher participation.

Although teacher response rates were not directly targeted in the enhanced approach as these were very high for cohort 1 (98.7%), we also found an increase in teachers’ response rate in the enhanced condition (99.3%), but teacher surveys were returned at a slower rate and more often required a reminder. The high parent response rate achieved in the enhanced condition resulted in individual teachers being asked to complete surveys for more children. The slower return rate and greater number of reminders required of teachers suggests this increased the burden on teachers, but did not prevent them from participating. Overall, while the enhanced approach fell short of our target of achieving complete parent and teacher screening data on 70% of Grade 1 students, it was substantially improved over the rate achieved in the standard condition (67% versus 55%).

It was also hypothesised that the enhanced research strategy would lead to the recruitment of a more representative sample of the population and the results were mixed. Participants in both cohorts did not differ from non-participants with respect to child age. In the enhanced research condition, there was increased participation of parents of female children, with cohort 2 participants more closely reflecting the gender balance in the community. This suggests that the enhanced research condition led to improvements in gender representativeness in the sample. The finding that a more socially advantaged sample participated in both cohorts suggests that regardless of the strategies used to improve response rates, some bias remained. It is possible that there are particular barriers to recruiting socially disadvantaged families to participate in school-based research, such as school non-attendance. This represents an important design challenge in recruiting a sample that is truly representative of the community and requires further study.

The results of this study demonstrate support for the strategies previously identified as effective in enhancing response rates. In line with Wolfenden et al. (2009), we found that promoting the research to school principals, teachers, parents was effective. Furthermore, follow-up reminder contacts to parents who had not made a decision regarding participation was crucial in boosting response rates, in line with other studies noting the importance of follow-up reminders [11]. The findings of this study emphasise the importance of having a multi-level approach, targeting multiple stakeholders (Education Department staff, principals, teaching staff, administrative staff and parents), in order to impact parent response rates in schools [2].

A limitation of our comparison between the enhanced and standard conditions was the use of the same group of schools. We cannot exclude the possibility that familiarity with study procedures and the longer established relationship with the study team may have also contributed to improved response rates. The simultaneous introduction of a range of additional strategies for cohort 2 precluded the possibility of identifying the relative effectiveness of specific strategies on parent response rates. However, the value of such comparisons may be limited. Single strategies may vary in their effectiveness across different social, economic or national contexts, and researchers typically employ as many strategies as they can afford within their practical, time and budgetary constraints. The multi-level approach used in this study requires high levels of engagement between researchers and school staff, making them most suitable for application in study designs where researchers have the resources to engage with multiple stakeholders. The paper-based survey methods used in this study are increasingly being replaced by electronic surveys, where the response rate challenges are likely to differ. Nevertheless, many of the strategies outlined are suitable across research contexts. A key message from this study is that the use of strategies that engage all levels of the school community in the business of sample recruitment/data collection, informed through consultation with the target participants, can produce significant improvements in parent response rates, with financial and time savings, and without any obvious adverse effects on the characteristics of the recruited sample and their intentions for long term participation.

## Conclusions

In conclusion, our findings indicate that implementing relatively simple enhancements that seek to promote awareness of and engagement in the study across all levels of the school community can result in significant improvements in parent participation, leading to more cost-effective recruitment and a more gender representative sample. This paper provides a detailed description of the multi-level strategies used to improve parent response rates in a large community-based study to act as guide for other researchers conducting school-based recruitment.

## Supporting Information

S1 Raw Data (XLS)
